# Case Report: Complete Response to Nivolumab in a Patient With Programmed Cell Death 1 Ligand 1-Positive and Multiple Gene-Driven Anaplastic Lymphoma Kinase Tyrosine Kinase Inhibitor-Resistant Lung Adenocarcinoma

**DOI:** 10.3389/fimmu.2021.686057

**Published:** 2021-11-04

**Authors:** Wen Dong, Pengfei Lei, Xin Liu, Qin Li, Xiangyang Cheng

**Affiliations:** ^1^ Department of Respiratory Medicine, Hainan Cancer Hospital, Haikou, China; ^2^ Department of Cardiothoracic Surgery, Yueyang Second People’s Hospital, Yueyang, China; ^3^ Department of Medical Center, Geneplus-Beijing Institution, Beijing, China; ^4^ State Key Laboratory of Respiratory Disease, National Clinical Research Center for Respiratory Disease, Guangzhou Institute of Respiratory Health, The First Affiliated Hospital of Guangzhou Medical University, Guangzhou, China

**Keywords:** lung cancer, EML4-ALK, nivolumab, complete remission, PD-L1

## Abstract

Multiple gene-driven programmed cell death 1 ligand 1 (PD-L1)-expressing non-small-cell lung cancer (NSCLC) is very rare. Previous studies have shown that patients with NSCLC with anaplastic lymphoma kinase (*ALK*) gene rearrangement rarely benefit from PD-L1 inhibitors. Besides the secondary mutations in *ALK* gene, other mechanisms might contribute to tumor resistance to ALK tyrosine kinase inhibitors (ALK-TKIs). Herein, we present a case of PD-L1-overexpressing lung adenocarcinoma that harbors both *EML4-ALK* gene rearrangement and *BRAF* mutation. In particular, a second molecular analysis after resistance to first- and second-generation ALK-TKIs revealed a high PD-L1 expression and tumor mutation burden. Therefore, treatment with nivolumab monotherapy, an anti-PD-1 inhibitor, was started and the patient achieved complete remission. This case report suggested that PD-1 inhibitors might be an effective treatment option for patients with multiple gene-driven PD-L1-expressing NSCLC harboring *ALK* gene rearrangement.

## Introduction

Next-generation sequencing has revealed new mechanisms that might contribute to drug resistance to anaplastic lymphoma kinase tyrosine kinase inhibitors (ALK-TKIs) in patients with non-small-cell lung cancer (NSCLC) with *ALK* gene rearrangement. These mechanisms include secondary *ALK* mutations and activation of bypass and downstream signaling pathways (1). Different strategies have been applied to overcome crizotinib resistance, including the use of second and third-generation ALK-TKIs for tumors with secondary *ALK* mutations, and corresponding targeted drugs to address drug resistance caused by activation of bypass or downstream signaling (1). However, additional complex drug resistance mechanisms have not been reported on a large scale; therefore, no standard treatment strategies exist for patients with such resistance mechanisms. Herein, we present a 37-year-old patient with NSCLC with both *ALK* gene rearrangement and *BRAF* mutation detected at the time of diagnosis. Programmed cell death 1 ligand 1 (PD-L1) overexpression and bypass and downstream pathway-activating mutations were acquired after disease progression with crizotinib and ceritinib treatments. The patient was switched to nivolumab monotherapy and finally achieved complete remission.

## Case Description

**Figure 1:**
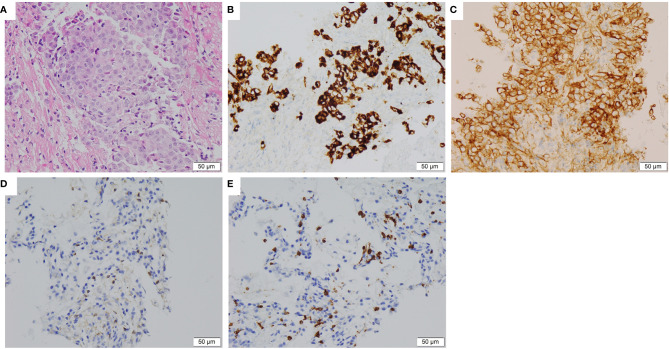
Histopathological and immunohistochemical staining of the lung tumor tissue sample. **(A)** Hematoxylin and eosin staining showing a poorly differentiated adenocarcinoma of primary biopsy (400×). **(B)** IHC staining showed ALK overexpression in 95% tumor cells of primary biopsy (400×). **(C)** IHC staining showed PD-L1 expression with Tumor Proportion Score 60% of primary biopsy (400×). **(D, E)** IHC staining CD4 and CD8 expression on T cells of rebiopsy showed that CD8 was positive (about 10%), and CD4 was only infiltrated individually (400×). IHC, immunohistochemical; ALK, anaplastic lymphoma kinase; PD-L1, Programmed cell death 1 ligand 1.

**Figure 2:**
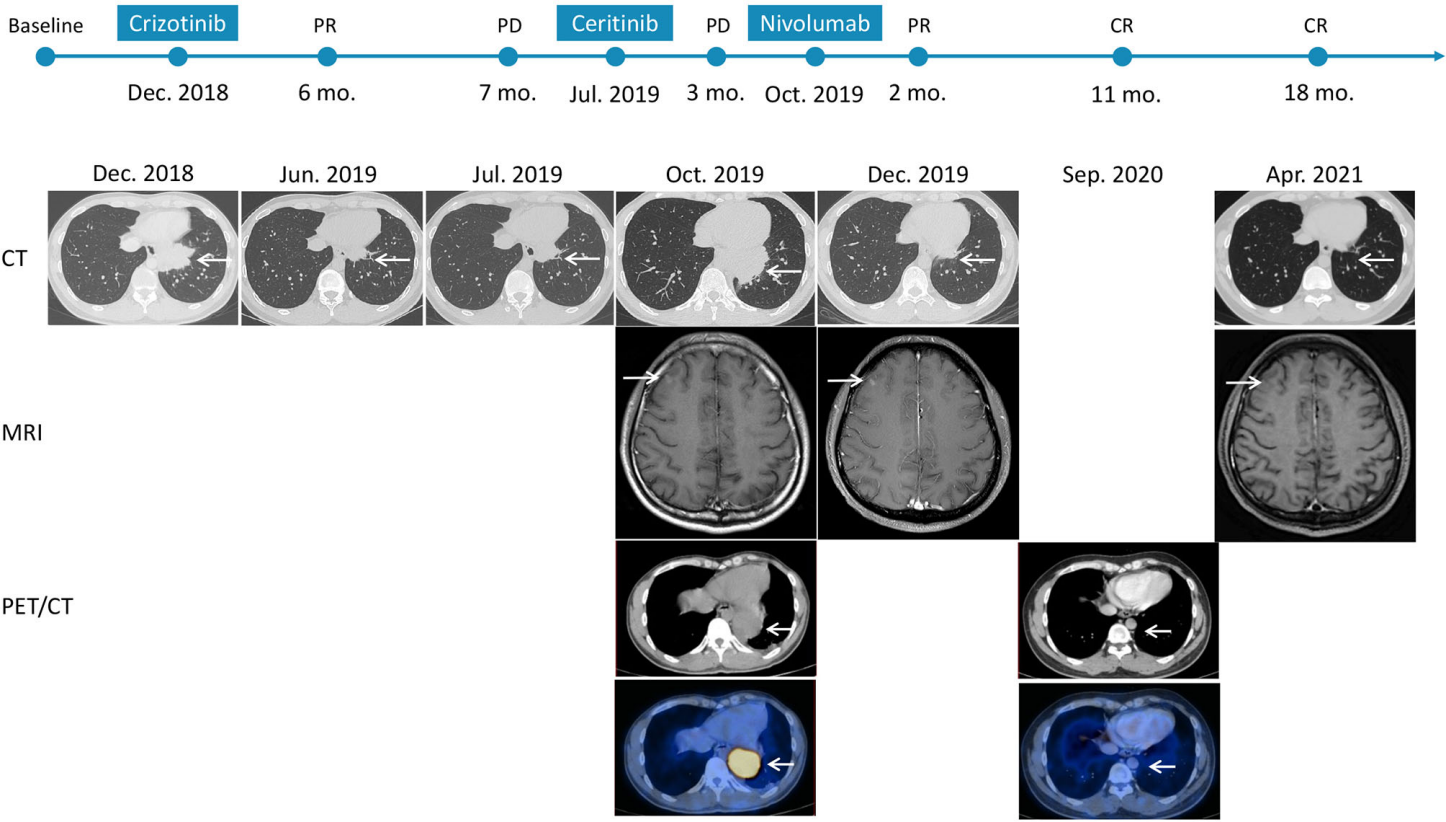
Time course depicting treatment process and radiological evaluation from 2018 to 2021. The PFS of crizotinib and ceritinib were 7 months and 3 months, respectively. And the duration of nivolumab was 18 months at the time of this report, and the patient is at present still on treatment and is receiving the drug. PR, partial response; PD, progressive disease; CR, complete response; CT, computed tomography; MRI, magnetic resonance imaging; PET/CT, positron emission tomography/computed tomography.

A 37-year-old non-smoker patient presented to our department with cough, sputum, and shortness of breath in December 2018. Computed tomography (CT) revealed a mass in the anterior inner basal segment of the left lower lobe with obstructive pneumonia. Lymph node metastasis was also found in the left hilar and mediastinal regions, and pericardial infiltration could not be excluded. Biopsy of the left lower lobe mass suggested a diagnosis of poorly differentiated adenocarcinoma (**Figure 1A**), and immunohistochemistry (IHC) showed positive expression of ALK (by VENTANA D5F3, Ventana Medical Systems, Inc., Oro Valley, AZ) and PD-L1 (Tumor Proportion Score, TPS=60%, by 22C3 pharmDx assay, Dako, Carpinteria, CA, USA) (**Figures 1B, C**). The initial DNA-based next-generation sequencing revealed echinoderm microtubule-associated protein-like 4 (*EML4*)*-ALK* fusion (v3), *BRAF* G466A, *PIK3CA* V344M mutation, and high tumor mutation burden (TMB, 13 Muts/Mb) in the plasma. The patient was finally diagnosed with left lung adenocarcinoma with multiple lymph node metastases (T2N3M0, Stage IIIb) and was treated with crizotinib, the first-generation ALK-TKI, 250 mg twice daily from December 2018. Treatment efficacy was evaluated as a partial remission in June 2019, while CT re-examination in July 2019 indicated an enlarged left lung mass, suggesting disease progression. Therefore, the patient was switched from crizotinib to a second-generation ALK-TKI ceritinib. However, 3 months later, CT showed a continued growth of the primary tumor as well as pericardial infiltration. In addition, simultaneous brain magnetic resonance imaging (MRI) suggested a right frontal lobe metastasis. A needle rebiopsy was performed on the patient’s left lower lung lesion, along with a molecular analysis, ALK and PD-L1 detection. Results revealed a new *KRAS* G12D mutation, the EML4-ALK fusion was not detected in DNA analysis, but ALK positive expression was seen in IHC. The mutation frequencies of all gene mutations were less than 5% and close to each other (**Supplementary Table 1**). Additionally, the TMB was still high (14.4 Muts/Mb), and PD-L1 expression increased compared to the pre-treatment values (TPS=90%). IHC assay of CD4 (clone: B468A1, diluted at 1:200, Santa Cruz, Texas, USA) and CD8 (clone 144B, diluted at 1:100, Abcam, Cambridge, UK) expression on T cells showed that CD8 was positive (about 10%), and CD4 was only infiltrated individually (**Figures 1D, E**). Finally, the patient was switched to PD-1 inhibitor nivolumab monotherapy because he declined chemotherapy. In December 2019, after four cycles of nivolumab treatment, the primary lung lesion and metastatic lymph nodes almost disappeared, and the intracranial lesion decreased in size. Positron emission tomography-CT (PET-CT) showed that all target lesions disappeared in September 2020. Therefore, treatment efficacy was evaluated as complete remission (CR), according to the Response Evaluation Criteria in Solid Tumors version 1.1 (RECIST 1.1). The latest follow-up was in April 2021, suggesting that the patient remained in CR for 7 months and was under continuous treatment with nivolumab. No obvious toxic and side effects were observed during the treatment. The treatment process and radiological evaluation were summarized in **Figure 2**.

## Discussion

Acquired resistance to crizotinib, a first-generation ALK inhibitor, usually emerges one year after treatment onset (2). Previous studies have shown that secondary *ALK* mutations and bypass or downstream pathway mutations in *EGFR*, *KRAS*, *ErbB*, *PIK3CA*, and *MET* contribute to acquired ALK-TKI resistance (1, 3). Second- and third-generation ALK-TKIs have been developed to overcome drug resistance conferred by ALK kinase domain mutations (4, 5). This patient received crizotinib and ceritinib treatments sequentially; however, his progression-free survival (PFS) was less than one year. Although EML4-ALK fusion existed before and after ALK-TKIs treatment, it is worth noting that there were many other bypass and downstream pathway activation mutations at the same time. *BRAF* mutations exist in 1–3% of patients with lung cancer. The *BRAF* G466A mutation in the protein kinase domain has been reported in colorectal, lung, and skin cancers. Studies have shown that this mutation can lead to impaired kinase activity; however, it can activate the mitogen-activated protein kinase kinase-extracellular signal-regulated kinase (MEK/ERK) signaling *via* RAF1 (2, 6). We speculated that *BRAF* and *PIK3CA* mutations might lead to the continuous activation of downstream signaling pathways, which could explain the short PFS. Moreover, after disease progression, along with acquiring a new *KRAS* mutation might lead to continuous activation of the downstream signaling pathways, which might explain the disease progression after switching to ceritinib. In addition, activation of the phosphoinositide 3-kinase (PI3K) signaling pathway can lead to PD-L1 upregulation (7), which has been shown as a major contributor to tumor immune escape (8) and might also explain tumor progression in this patient after ALK-TKI treatment.

Previous studies have shown that *ALK* rearranged NSCLC patients had low PD-L1 expression and low CD8+ tumor infiltrating lymphocytes (7–9), and TMB is low with fewer non-synonymous mutations (10). In addition, retrospective studies have shown lack of efficacy with single-agent PD-1/PD-L1 inhibitor in *ALK* rearranged NSCLC (7, 11–13). However, immunotherapy has shown considerable efficacy in patients with *KRAS* or *BRAF* non-V600 mutations (11, 14). Since the mutation frequencies of all gene mutations were less than 5% and close to each other, it is difficult to analyze the clonal and subclone distribution of these gene alterations, including ALK. The patient in this report successful benefited from nivolumab may be due to multiple factors rarely co-occurring with *ALK* rearrangement, including high expression of PD-L1, positive CD8+ tumor infiltrating lymphocytes, high TMB, and co-mutation with *KRAS* and *BRAF*.

In conclusion, ALK positive NSCLC with multiple driving mutations, high TMB, PD-L1 overexpression, and CD8+ tumor infiltrating lymphocytes is very rare. The selection of an appropriate therapeutic strategy is very important for this group of patients. The case presented here showed that tumor gene mutations changed with disease progression, suggesting the necessity of large-panel genetic tests in the process of targeted therapy for patients with NSCLC, especially those with disease progression. Moreover, the high efficacy of nivolumab suggested that PD-L1 inhibitors might be a good treatment option for these patients.

## Data Availability Statement

The datasets presented in this study can be found in online repositories. The names of the repository/repositories and accession number(s) can be found in the article/**Supplementary Material**.

## Ethics Statement

The studies involving human participants were reviewed and approved by Institutional Review Board of the First Affiliated Hospital of Guangzhou Medical University (Guangzhou, Guangdong, China). The patients/participants provided their written informed consent to participate in this study.

## Author Contributions

XC conceptualized and designed the study. WD and PL collected, processed, and analyzed the data. XL and QL drafted the manuscript and figures. All authors contributed to the article and approved the submitted version.

## Conflict of Interest

The authors declare that the research was conducted in the absence of any commercial or financial relationships that could be construed as a potential conflict of interest.

## Publisher’s Note

All claims expressed in this article are solely those of the authors and do not necessarily represent those of their affiliated organizations, or those of the publisher, the editors and the reviewers. Any product that may be evaluated in this article, or claim that may be made by its manufacturer, is not guaranteed or endorsed by the publisher.
